# Silicate Urolithiasis during Long-Term Treatment with Zonisamide

**DOI:** 10.1155/2013/629381

**Published:** 2013-07-02

**Authors:** Satoru Taguchi, Yorito Nose, Toshikazu Sato, Teruaki Kobayashi, Kanami Takaya, Akira Ishikawa, Yukio Homma

**Affiliations:** ^1^Department of Urology, Tokyo Teishin Hospital, Tokyo 102-8798, Japan; ^2^Department of Urology, Graduate School of Medicine, The University of Tokyo, Tokyo 113-8655, Japan; ^3^Department of Laboratory Medicine, Tokyo Teishin Hospital, Tokyo 102-8798, Japan; ^4^Department of Hospital Pharmacy, Tokyo Teishin Hospital, Tokyo 102-8798, Japan

## Abstract

Silicate urinary calculi are rare in humans, with an incidence of 0.2% of all urinary calculi. Most cases were related to excess ingestion of silicate, typically by taking magnesium trisilicate as an antacid for peptic ulcers over a long period of time; however, there also existed unrelated cases, whose mechanism of development remains unclear. On the other hand, zonisamide, a newer antiepileptic drug, is one of the important causing agents of iatrogenic urinary stones in patients with epilepsy. The supposed mechanism is that zonisamide induces urine alkalinization and then promotes crystallization of urine components such as calcium phosphate by inhibition of carbonate dehydratase in renal tubular epithelial cells. Here, we report a case of silicate urolithiasis during long-term treatment with zonisamide without magnesium trisilicate intake and discuss the etiology of the disease by examining the silicate concentration in his urine.

## 1. Introduction

Silicate urinary calculi are rare in humans, with an incidence of 0.2% of all urinary calculi [1]. Most cases were related to excess ingestion of silicate, typically by taking magnesium trisilicate as an antacid for gastric and duodenal ulcers over a long period of time [2, 3]. However there also existed unrelated cases, which might be affected by other factors [4]. 

On the other hand, zonisamide, a newer antiepileptic drug, is one of important causing agents of iatrogenic urinary stones in patients with epilepsy. The supposed mechanism is that zonisamide induces urine alkalinization and then promotes crystallization of urine components such as calcium phosphate by inhibition of carbonate dehydratase in renal tubular epithelial cells [5]. 

To our knowledge, there has been no report of silicate urolithiasis supposed to be associated with zonisamide. Here, we report a case of silicate urolithiasis during long-term treatment with zonisamide for epilepsy and discuss the etiology of the disease by examining the silicate concentration in his urine. 

## 2. Case Presentation

**Figure 1:**
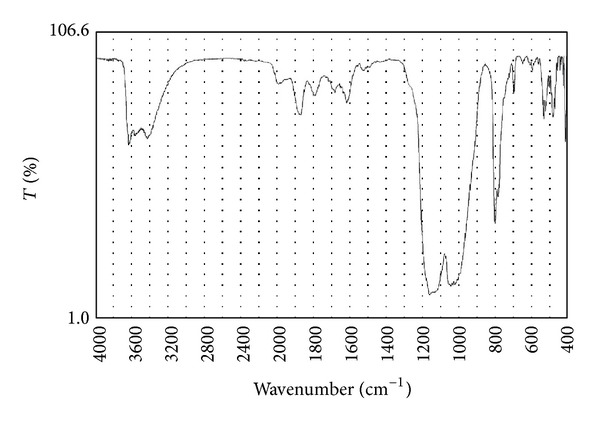
Infrared spectrophotometry revealed that the calculus was comprised of over 98% of silicon dioxide.

A 30-year-old male visited a general internal medicine clinic with a complaint of left back pain and was diagnosed with ureteral lithiasis by a computed tomographic scan. He was introduced to our hospital for further treatment; however, he had already expelled the stone at the initial visit. A computed tomographic scan we performed presented no other urinary calculi. The urinalysis revealed a pH of 7.0 and the examination of urinary sediment showed 1–4 WBC and 1–4 RBC per high-power field. Peripheral blood count and blood chemistry were unremarkable except for high uric acid level (7.8 mg/dL). The stone he brought with was black, round, smooth, and 3 mm in diameter. On infrared spectrophotometry, its wavelength pattern exhibited a peak at 1100 cm^−1^, which indicated that it consisted of over 98% silicon dioxide (SiO_2_) (Figure 1). While he had no history of taking magnesium trisilicate, he had been taking 600 mg per day of valproic acid for the treatment of epilepsy from 14 years of age and taking 200 mg per day of zonisamide in addition from 19 years of age due to poor control. We examined the silicate concentration in his urine, and the result was 3.25 mg/dL as SiO_2_. 

## 3. Discussion

Urinary silicate calculi are common in herbivorous animals such as ovine and cattle but rare in humans with an incidence of 0.2% of all urinary calculi [1, 6]. In Japan, 50 patients with urinary silicate calculi have been reported in the literature [4, 6–10]. All patients except a 10-month-old infant case were adults aged between 24 and 77 years. As shown in the literature, urinary silicate calculi tend to occur in patients taking large amounts of antacids containing silicate, such as magnesium trisilicate [1–3]. However, the prevalence of magnesium trisilicate was 61.5% in Japan [9], and the other cases might be affected by different factors. 

Our case had no history of taking magnesium trisilicate; however, he had been taking zonisamide for more than 10 years. Carbonic anhydrase inhibitors such as acetazolamide, topiramate, and zonisamide are well known to cause iatrogenic urinary stones. The supposed mechanism of their stone formation is that they induce urine alkalinization and then promote crystallization of urine components such as calcium phosphate by inhibition of carbonate dehydratase in renal tubular epithelial cells [5]. According to Takemoto, the trigger of silicate stone formation is also urine alkalinization [6]. He assumed that deposition of the calcium salt around silicate gel might be driven by urine alkalinization, after high concentration of the silicate in the urine induced aggregation of the silicate [6]. The urinary pH of patients with silica stones has been mentioned but twice in the English literature: it was 7.5 in the first instance [7], and 7.5 in the second where 6 months after discontinuing the antacid, it dropped to 5.5 [11]. Our case also had a relatively alkaline pH of 7.0. 

The important factor other than alkalinization must be the urine concentration of silica. As of now, there have been few studies concerning normal silicate concentration in human urine, and furthermore, there has been only one case with silicate urolithiasis in whom the level of urinary silica was determined. Haddad reported that his patient with silicate urolithiasis had 6 mg/dL [1], while random sample of human urine contains an average of 1.31 mg [12] to 1.46 mg/dL [2]. According to Page, these figures were proportional to the intake of silica-containing foods such as vegetables, grains, or sea food; however, no figures were given [2]. Page also reported that volunteers who received 5 g of magnesium trisilicate per day excreted in the urine 5.2% of the ingested silica. In these volunteers, the urinary silica went up from a pretreatment level of 1.46 mg/dL to 12.27 mg/dL [2]. This elevation was apparent on the second day of administration. The urinary silica decreased to 1.8 mg/dL on the second day after discontinuation of the magnesium trisilicate [2]. Our patient had 3.25 mg/dL, which is 3 times the normal. The reason why his urinary silica increased remains unclear. Kinoshita mentioned that many tablets contain aluminum magnesium silicate or colloidal silicon dioxide derived from excipients in their manufacturing process, such as binders, lubricants, absorbents, and emulsion stabilizers, and that they might cause a formation of silicate stones [10]. Since both zonisamide and valproic acid which our patient had been taking for years contain such silicon compounds, they might contribute to the increase of his urinary silica. 

In summary, concerning our case, zonisamide might contribute to silicate stone formation in two respects: alkalinization and increase of silicate concentration in urine. To our knowledge, this is the first case report of silicate urolithiasis supposed to be associated with zonisamide.
